# Thermal Stability of Color Centers in Lithium Fluoride Crystals Irradiated with Electrons and N, O, Kr, U Ions

**DOI:** 10.3390/ma18194441

**Published:** 2025-09-23

**Authors:** Zhadra Malikova, Zhakyp T. Karipbayev, Abdirash Akilbekov, Alma Dauletbekova, Anatoli I. Popov, Vladimir N. Kuzovkov, Ainash Abdrakhmetova, Alyona Russakova, Muratbek Baizhumanov

**Affiliations:** 1 Institute of Physical and Technical Sciences, L.N. Gumilyov Eurasian National University, Munaitpasov Str. 13, Astana 010008, Kazakhstan; malikova_zhb_1@enu.kz (Z.M.); akilbekov_at@enu.kz (A.A.); dauletbekova_ak@enu.kz (A.D.); abdrakhmetova_aa@enu.kz (A.A.); baizhumanov_mzh_13.1@enu.kz (M.B.); 2 Institute of Solid State Physics, University of Latvia, Kengaraga 8, LV-1063 Riga, Latvia; kuzovkov@latnet.lv; 3 National Scientific Laboratory for Collective Use, Sarsen Amanzholov East Kazakhstan University, 34 Tridtsatoy Gvardeiskoy Divizii Str., Ust-Kamenogorsk 070002, Kazakhstan; arusakova@ektu.kz

**Keywords:** LiF, Frenkel defects, swift heavy ions, thermal stability, optical absorption

## Abstract

Lithium fluoride (LiF) crystals are widely employed both as optical windows transparent in the ultraviolet spectral region and as efficient personal dosimeters, with their application scope recently expanding into lithium-ion technologies. Moreover, as an alkali halide crystal (AHC), LiF serves as a model system for studying and simulating radiation effects in solids. This work identifies radiation-induced defects formed in lithium fluoride upon irradiation with swift heavy ion beams (N, O, Kr, U) and intense pulsed electron beams, investigates their thermal stability, and performs computer modeling of annealing processes. The theoretical analysis of existing experimental kinetics for F-centers induced by electron and heavy ion irradiation reveals considerable differences in the activation energies for interstitial migration. A strong correlation between the activation energy $E_{a}$ and the pre-exponential factor X($E_{a}$) is observed; notably, X($E_{a}$) is no longer constant but closely matches the potential function Ea. Indeed, with increasing irradiation dose, both the migration energy $E_{a}$ and pre-exponential factor X decrease simultaneously, leading to an effective increase in the defect diffusion rate.

## 1. Introduction

For a long time, lithium fluoride (LiF) crystals have been utilized as optical windows for the ultraviolet spectral region and as dosimetric materials in personal dosimeters designed for detecting gamma radiation, electrons, and thermal neutrons. Consequently, electron-hole and excitonic processes responsible for storing the absorbed radiation energy have been extensively studied in these crystals. The thermally stimulated luminescence (TSL) peak in the temperature range of 300–550 K serves as a measure of the absorbed dose. Their high level of tissue equivalence has led to the development of effective personal dosimeters [1]. Recently, the application domain of LiF has expanded to include lithium-ion batteries, lithium-based optical and laser materials, and advanced materials for nuclear applications [2,3,4,5,6,7,8,9,10,11,12,13,14,15]. The applications and future prospects of LiF have been thoroughly discussed in recent reviews, along with extensive references therein [16,17,18]. Furthermore, as an alkali halide crystal (AHC), LiF constitutes a model system for the investigation and simulation of radiation effects in solids [1,16,17,18]. Particle irradiation (neutrons, electrons, ions) generates radiation-induced defects therein, often leading to a progressive degradation of its functional properties [17,18,19,20,21,22,23,24,25,26]. Swift heavy ion (SHI) irradiation of alkali halides, as well as binary and complex oxides, induces not only isolated *F*-centers but also complex aggregate *F*-type centers (*F*_t_, t = 2, 3, 4) [27,28,29,30,31,32,33,34,35,36,37,38,39,40,41,42]. The optical absorption band maxima of these electron centers are listed in Table 1.

Currently, the luminescent and optical properties of *F*-type defects induced in lithium fluoride (LiF) under prolonged irradiation by ions of various masses and energies have been extensively investigated [17,28,30,31,33,40,41,42,43]. The significance of this topic arises from the widespread application of ion beams in science and technology, such as modifying the surface layers of functional materials.

It is widely accepted today that, besides the collision (knock-on) mechanism, another mechanism involving the excitation and ionization of the electronic subsystem by particles or quanta occurs, resulting in the formation of elementary electronic excitations such as excitons and electron-hole pairs. These electronic excitations relax to self-trapped states ($e_{s}$), subsequently producing Frenkel pairs ((F−H), (α−I)), which constitute primary color centers. In alkali halide crystals (AHCs), and specifically in LiF, the excitonic defect formation mechanism is significantly more effective than the elastic collision mechanism [44,45,46,47,48,49,50]. Furthermore, under heavy ion irradiation, the excitonic mechanism is also dominant [33,51].

Radiation-induced defect formation in AHCs subjected to heavy-ion irradiation strongly depends on energy loss, irradiation temperature, and fluence (dose). Irradiation to high fluences leads to the formation of more complex aggregate electronic defects, accompanied by a decrease in the concentration of F and $F_{n}$ centers [40]. Irradiation with ions having energy losses above the critical threshold of approximately 10 keV/nm results in track core damage and initiation of dislocations in individual ion tracks. Upon overlapping of these tracks, the enlargement and ordering of dislocation loops occur, leading to the formation of nanostructures [17].

Heavy ions with velocities corresponding to energy losses of approximately 10 keV/nm induce core damage within tracks and initiate dislocations in individual tracks; as these tracks overlap, dislocation loops enlarge and become ordered, facilitating nanostructure formation [40,43,44]. It has been demonstrated that ion-induced damage significantly affects mechanical properties due to the reduced mobility of dislocations and the modification of nanostructures and ion-induced stresses [40,44].

As previously noted, radiation-induced defects in LiF affect its functional properties. To restore the original properties of LiF single crystals, thermal treatments must be carried out, during which radiation-induced defects lose their stability and recombine. Thus, it is crucial to investigate not only the processes of radiation-induced defect formation but also their thermal stability and the annealing mechanisms.

The aim of this study is to identify radiation-induced defects formed in lithium fluoride upon irradiation with swift heavy ion beams (N, O, Kr, U) and high-power pulsed electron beams, to investigate the thermal stability of these defects, and to perform computer simulations of the annealing processes.

## 2. Experimental and Theoretical Methods of Research

For the present study, high-purity LiF single crystals grown at JSC “INKROM” (GOI Vavilov, St. Petersburg) were used. The transparency edge of the LiF crystals is at 11.5 eV, indicating their high quality. The crystals were irradiated with electron beams and with ions of 28 MeV ^16^O, 23 MeV ^14^N, 150 MeV ^84^Kr, and 2640 MeV ^238^U to various fluences at room temperature (RT). Irradiation with 28 MeV ^16^O, 23 MeV ^14^N, and 150 MeV ^84^Kr ions was carried out at the DC-60 cyclotron (Astana, Kazakhstan), while irradiation with 2640 MeV ^238^U ions was performed at the GSI accelerator facility (Darmstadt, Germany).

Additionally, pulsed electron beams with a pulse duration of 10 ns and an average electron energy of 250 keV were used for irradiation. The energy density per pulse was 15 $\frac{mJ}{cm^{2}}$. The electron penetration depth was 0.2 mm. The absorbed dose per single pulse in the LiF crystal was $8\times 10^{2}$ Gy. Electron irradiation was conducted using a pulsed electron spectrometer (Astana, Kazakhstan).

The parameters of the 28 MeV ^16^O, 23 MeV ^14^N, 150 MeV ^84^Kr, and 2640 MeV ^238^U ions were calculated using the SRIM code [52] and are presented in Table 2.

The maximum energy of an electron ejected during the interaction of an ion with a solid is determined by the following expression:

(1) $$E_{max}^{e}=\frac{{4m}_{e}E_{ion}}{M},$$

where $m_{e}$ is the electron mass, $E_{max}^{e}$ is the ion energy, and *M* is the ion mass.

The calculated maximum electron energies $E_{max}^{e}$ for oxygen ions (^16^O) is 3.84 keV, for nitrogen ions (^14^N) is 3.61 keV, for krypton ions (^84^Kr) is 3.91 keV, and for uranium ions (^238^U) is 24.34 keV. The maximum energy of electrons ejected by ions, based on scaling factors, for ^16^O is $E_{max}^{e}$ = 22 × 1.75 = 38.5 keV; for ^14^N: $E_{max}^{e}$ = 22 × 1.65 = 36.1 keV; for ^84^Kr: $E_{max}^{e}$ = 22 × 1.79 = 39.38 keV; and for ^238^U: $E_{max}^{e}$ = 22 × 11.09 = 244 keV.

These primary electrons, upon further interactions within the solid, can produce secondary or so-called δ-electrons. For these ions, the energy of the δ-electrons is typically less than 50 eV, and the free path of such low-energy electrons is approximately 1 nm. According to electron energy-loss spectra, δ-electrons are capable of generating anionic excitons, separated electrons and holes, plasmons, and cationic excitons.

It should be noted that electronic energy losses are dominant for ^16^O, ^14^N, and ^84^Kr ions. The ratio of electronic to nuclear stopping powers is 1354, 1382, and 551, respectively, which is sufficiently large that the displacement of lattice ions (${Li}^{+}$, $F^{-}$) by the incoming ion can be neglected. For uranium ions, this ratio is 12, and thus, nuclear (collision) processes cannot be ignored in defect formation.

Optical absorption spectra were measured in the energy range of 1.5–6.5 eV using a SPECORD 250 UV VIS spectrophotometer (Analytik Jena, Germany) and a PERSEE T8DCS spectrophotometer (Beijing, China). Spectra were recorded after irradiation at room temperature (RT).

Stepwise annealing of the irradiated LiF crystals was carried out in a muffle furnace–SNOL (Vilnus, Lithuania), on an aluminum substrate, in air. After cooling the samples down to room temperature, absorption spectra were measured. These multiple “heating–cooling–measurement” cycles were performed under identical conditions at a heating rate of 5 K per second. All presented spectra were measured at room temperature (RT).

Theoretical modeling was performed on a supercomputer at the Institute of Solid State Physics, University of Latvia (ISSP UL). The Latvian Supercluster (LASC) has the following technical specifications: Linux (CentOS) operating system; total available resources include 2376 CPUs, with a theoretical peak performance of approximately 157 TFlops, 12 TB of RAM, and 127 TB of total disk storage. LASC is used for (a) computational modeling of advanced materials; (b) modeling and interpretation of experimental results.

## 3. Results and Discussion

### 3.1. Experimental Investigation

The thermal stability of various defects in LiF crystals irradiated with heavy ions depends on both the ion energy loss and the fluence [53,54,55,56,57]. During thermal treatment, two distinct processes occur: (1) the thermal decay of color centers, and (2) the diffusion of color centers or their decay products. Since hole-type color centers are thermally less stable than electron-type color centers, their decomposition with the formation of *H* centers and vacancies occurs at the initial stages of annealing. The generated *H* centers are mobile and recombine with electron-type color centers. Anion vacancies ($V_{a}^{+}$), which are more mobile than *F* centers, may also participate in the annealing process. The diffusion of $V_{a}^{+}$ and *F* centers can lead to the formation of complex electron-type color centers such as $F+F\to F_{2}$, $F+F_{2}\to F_{3}$, and etc. and larger aggregates [53].

**Figure 1:**
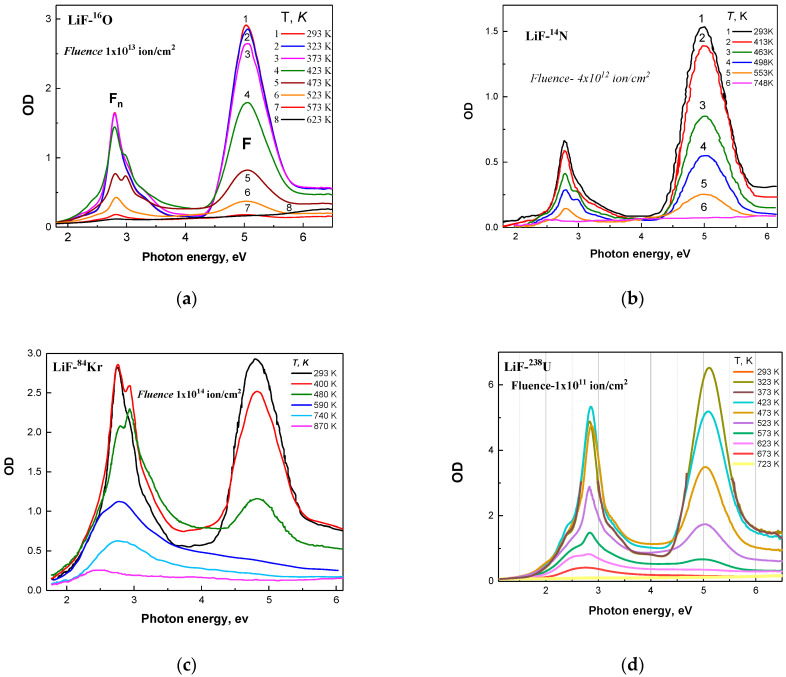
(**a**) Optical absorption (OA) spectra of LiF crystals irradiated with 28 MeV ^16^O ions to a fluence of $\Phi \;=\;1\times 10^{13}$ ions/cm^2^ after stepwise annealing in the range of 293–623 K; (**b**) OA spectra after irradiation with 23 MeV ^14^N ions to a fluence of $\Phi \;=\;4\times 10^{12}$ ions/cm^2^ and stepwise annealing in the range of 293–748 K; (**c**) OA spectra after irradiation with 150 MeV ^84^Kr ions to a fluence of $\Phi \;=\;1\times 10^{14}$ ions/cm^2^ and stepwise annealing in the range of 293–870 K; (**d**) OA spectra after irradiation with 2640 MeV ^238^U ions to a fluence of $\Phi \;=\;1\times 10^{14}$ ions/cm^2^ and stepwise annealing in the range of 293–723 K.

Figure 1 shows the optical absorption (OA) spectra of LiF crystals irradiated with 28 MeV ^16^O, 23 MeV ^14^N, 150 MeV ^84^Kr, and 2640 MeV ^238^U ions after stepwise heating to specific temperatures *T* in the range from 293 K to 870 K.

**Figure 2:**
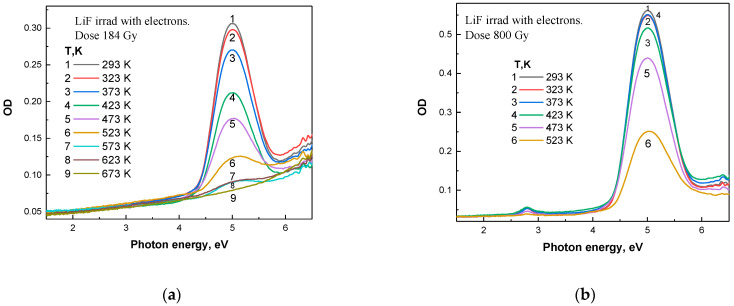
(**a**) Optical absorption (OA) spectra of LiF crystals irradiated with electrons to a dose of 184 Gy after stepwise annealing in the range of 293–673 K; (**b**) OA spectra of LiF crystals irradiated with electrons to a dose of 800 Gy after stepwise annealing in the range of 293–523 K.

Figure 2 presents the optical absorption spectra of LiF crystals irradiated with an electron beam to doses ranging from 184 Gy to 800 Gy at room temperature, after stepwise annealing to specific temperatures in the range of 293 K to 870 K.

### 3.2. Annealing Analysis of Defects

The integrated absorption of F and $F_{n}$ color centers were determined in the spectral ranges of 4.13–5.90 eV for F centers and 1.77–4.13 eV for $F_{n}$ centers according to Equations (2) and (3). The energy range for F and $F_{n}$ color centers is consistent for irradiation with ^84^Kr, ^14^N, ^16^O, and ^238^U ions, as well as for electrons at doses of 184 Gy and 800 Gy. Therefore, the following formulas were used to calculate the integral optical absorption values [29]:

(2) $$A_{F}=\int_{4.13}^{5.90}D\left(\varepsilon \right)\;d\varepsilon$$

(3) $$A_{Fn}=\int_{1.77}^{4.13}D\left(\varepsilon \right)\;d\varepsilon ,$$

where *D_F_* and $D_{Fn}$ are the optical densities at the absorption maxima of the *F* and $F_{n}$ centers, respectively, and *ε* is the photon energy (eV).

**Figure 3:**
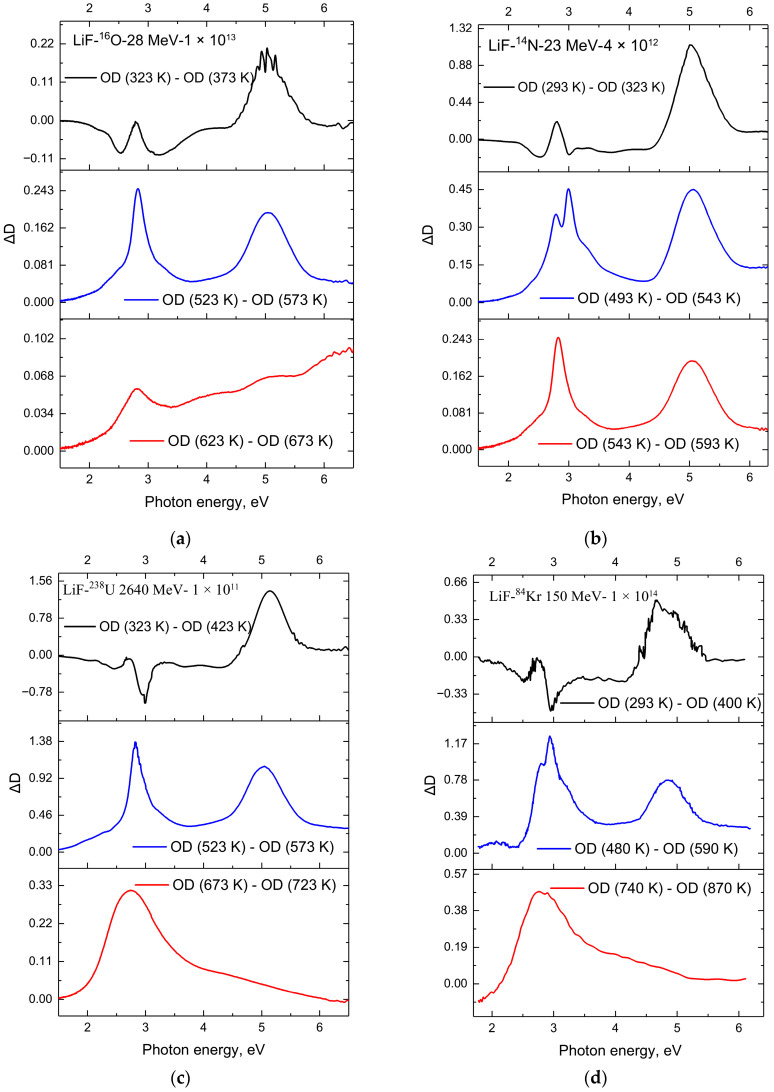
(**a**) Absorption spectra after thermal annealing of LiF crystals irradiated with 28 MeV ^16^O ions to a fluence of $\Phi \;=\;1\times 10^{13}$ ions/cm^2^, following stepwise annealing in the range of 293–623 K; (**b**) after irradiation with 23 MeV ^14^N ions to a fluence of $\Phi \;=\;4\times 10^{12}$ ions/cm^2^ and stepwise annealing in the range of 293–748 K; (**c**) after irradiation with 2640 MeV ^238^U ions to a fluence of $\Phi \;=\;1\times 10^{14}$ ions/cm^2^; (**d**) after irradiation with 150 MeV ^84^Kr ions to a fluence of $\Phi \;=\;1\times 10^{14}$ ions/cm^2^ and stepwise annealing in the range of 293–870 K.

**Figure 4:**
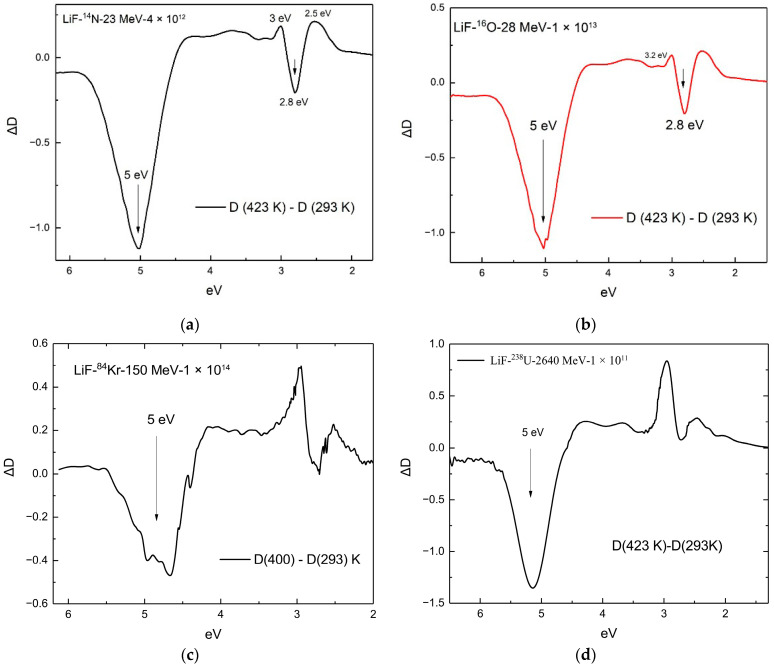
(**a**) Difference absorption spectra after thermal annealing of LiF crystals irradiated with 23 MeV ^14^N ions to a fluence of $\Phi \;=\;4\times 10^{12}$ ions/cm^2^, for the (423–293) K interval; (**b**) difference spectra for the (423–293) K interval after irradiation with 28 MeV ^16^O ions to a fluence of $\Phi \;=\;1\times 10^{13}$ ions/cm^2^; (**c**) difference spectra for the (400–293) K interval after irradiation with 150 MeV ^84^Kr ions to a fluence of $\Phi \;=\;1\times 10^{14}$ ions/cm^2^; (**d**) difference spectra for the (423–293) K interval after irradiation with 2640 MeV ^238^U ions to a fluence of $\Phi \;=\;1\times 10^{14}$ ions/cm^2^.

The optical absorption spectra at various annealing stages are shown in Figure 3 for samples (a) irradiated with 28 MeV ^16^O ions to a fluence of $\Phi \;=\;1\times 10^{13}$ ions/cm^2^; (b) irradiated with 23 MeV ^14^N ions to a fluence of $\Phi \;=\;4\times 10^{12}$ ions/cm^2^; (c) irradiated with 2640 MeV ^238^U ions to a fluence of $\Phi \;=\;1\times 10^{14}$ ions/cm^2^; and (d) irradiated with 150 MeV ^84^Kr ions to a fluence of $\Phi \;=\;1\times 10^{14}$ ions/cm^2^. The curves in Figure 4 indicate the spectral regions where absorption decreases or, conversely, increases during the annealing process. Analysis of these figures using Equations (2) and (3) allowed us to make the following assessments, as presented below.

Annealing at 400 K for LiF crystals irradiated with 150 MeV ^84^Kr ions to a fluence of $\Phi \;=\;1\times 10^{14}$ ions/cm^2^ leads to a reduction in *F* center absorption ($A_{F}\;$ = 12%) and a general increase in $F_{n}$($\frac{F_{2}}{F_{3}^{+}}$) centers ($A_{Fn}=\;$15%) (Figure 3c). The next step at 480 K results in a significant decrease in the integral absorption of *F* centers ($A_{F}$ = 32%); a decrease in $A_{Fn}$ by 7% is also observed (relative to 400 K). At 480 K, the absorption bands of $F_{3}^{+}$ and $F_{2}$ centers can be clearly distinguished (Figure 3c). $F_{3}^{+}$ and $F_{2}$ centers are annealed at around 590 K. Below 500 K, the primary process is the diffusion of *H* centers. With increasing temperature, *F* centers become mobile and form more complex $F_{n}$ centers according to the reaction $F_{n}$ + *F* → $F_{n}$ + 1. At annealing temperatures of around 600 K, impurity and lithium colloids are formed (Figure 3d). Complete annealing occurs at approximately 850 K.

Annealing at 323 K for LiF crystals irradiated with 28 MeV ^16^O ions to a fluence of $\Phi \;=\;1\times 10^{13}$ ions/cm^2^ results in little change in *F* center concentration (a decrease of $A_{F}$ = 4%), and also a reduction in $F_{n}$($\frac{F_{2}}{F_{3}^{+}}$) centers ($A_{Fn}\;$ = 6%). Annealing at 423 K for these samples leads to a decrease in *F* center absorption ($A_{F}$ = 33%) and an increase in $F_{n}$($\frac{F_{2}}{F_{3}^{+}}$) center absorption ($A_{Fn}$ = 6.83%) compared to the previous annealing at 373 K. At 623 K, *F* centers and $F_{n}$($\frac{F_{2}}{F_{3}^{+}}$) centers are almost completely annealed.

Annealing at 413 K for LiF crystals irradiated with 23 MeV ^14^N ions to a fluence of $\Phi \;=\;4\times 10^{12}$ ions/cm^2^ decreases the absorption of both *F* centers ($A_{F}$ = 24%) and $F_{n}$($\frac{F_{2}}{F_{3}^{+}}$) centers ($A_{Fn}$ = 15%) to a similar extent. The subsequent step at 463 K also reduces the absorption of *F* centers ($A_{F}$ = 23%) and, similarly, $F_{n}$($\frac{F_{2}}{F_{3}^{+}}$) centers ($A_{Fn}$ = 17%). At 463 K, the absorption peaks of $F_{3}^{+}$ and $F_{2}$ centers can be clearly resolved. $F_{3}^{+}$ and $F_{2}$ centers are annealed at approximately 590 K.

Annealing at 423 K for LiF crystals irradiated with 2640 MeV ^238^U ions to a fluence of $\Phi \;=\;1\times 10^{14}$ ions/cm^2^ results in a decrease in *F* center absorption ($A_{F}$ = 13%) and an increase in $F_{n}$($\frac{F_{2}}{F_{3}^{+}}$) centers ($A_{Fn}=\;$7%). At the next step of 473 K, both *F* center absorption ($A_{F}$ = 24%) and $F_{n}$($\frac{F_{2}}{F_{3}^{+}}$) center absorption ($A_{Fn}$ = 7.4%) decrease compared to 423 K. As in the case of Kr ion irradiation, the formation of more complex aggregates and colloids is observed. Almost complete annealing is observed at 723 K.

A similar analysis was carried out for LiF crystals irradiated with electron beams. Heating the crystals leads to the destruction of color centers. Figure 2a presents the results of thermal stability studies of accumulated color centers in LiF crystals irradiated with electron fluxes to a dose of 184 Gy. As in the case of ion-irradiated samples, the crystal was heated to elevated temperatures, held for 10 min, and then cooled to room temperature. The absorption spectra were measured at room temperature. Examination of the absorption spectra of LiF crystals irradiated with electron beams to a dose of 184 Gy revealed only the *F* center band. With increasing annealing temperature, the *F* centers are destroyed, while no $F_{n}$ centers are formed.

**Figure 5:**
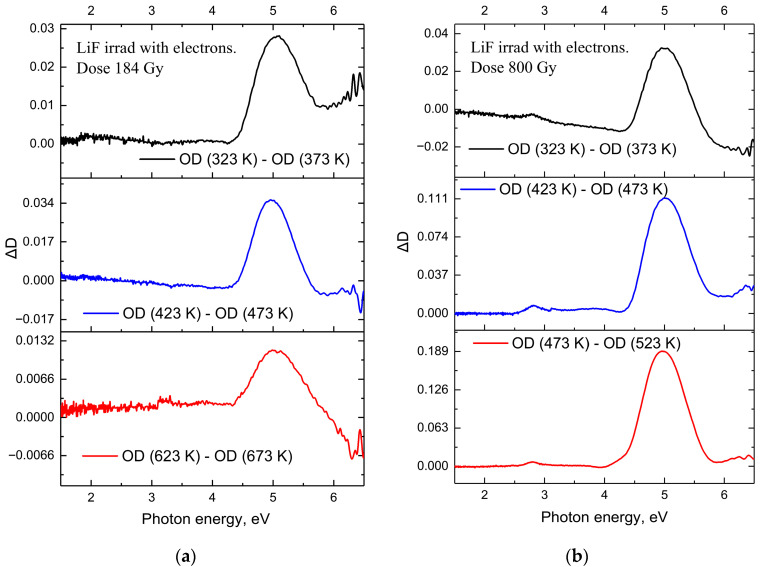
(**a**) Change in absorption (∆D) after thermal annealing of LiF crystals irradiated with electrons to a dose of 184 Gy, following stepwise annealing in the range of 293–673 K; (**b**) change in absorption after irradiation to a dose of 800 Gy and subsequent stepwise annealing in the range of 293–523 K.

After irradiation of LiF crystals with a pulsed electron beam to a dose of 800 Gy and subsequent stepwise annealing in the temperature range of 293–523 K, pronounced changes are observed in the optical absorption spectra, associated with the relaxation of radiation-induced defects—primarily *F* centers. In the absorption spectra (Figure 2b), the maximum intensity of the *F* center band is retained at the early stages of thermal treatment (up to ~400 K), indicating a high thermal stability of these defects at relatively low temperatures. However, with further increase in temperature, the band intensity decreases sharply, especially after annealing at 473 K and above, which indicates the thermal annihilation of *F* centers. This process is also clearly manifested in the differential spectra (Figure 5b), where the difference in optical density between consecutive annealing steps illustrates the decrease in the concentration of absorbing centers: the maximum in the *F* center region decreases significantly with increasing temperature, particularly in the range from 473 to 523 K. This behavior indicates that the most intensive relaxation of radiation defects occurs in this temperature range.

Annealing at 373 K for LiF crystals irradiated with electron fluxes to a dose of 800 Gy results in a significant decrease in *F* center absorption ($A_{F}$ = 7%) compared to the previous annealing temperature of 323 K. Further annealing at 473 K leads to a reduction in absorption by an additional $A_{F}$ = 32% relative to the value at 373 K, and at 523 K—up to 50%, indicating a substantial elimination of *F* centers.

A comprehensive analysis of the absorption spectra, their differential changes, and the integral intensity of the *F* center band demonstrates that the main processes of thermal relaxation of radiation defects in LiF crystals occur at temperatures above 470 K. It is in this range that a sharp decrease is observed in both the amplitude and the integrated intensity of the *F* center band, reflecting the efficient annealing of radiation defects formed as a result of electron irradiation.

From Figure 1a–d and Figure 2a,b, it is evident that the primary defects formed under SHI irradiation are *F* centers and $F_{n}$ centers (*n* = 2, 3, 4), as well as their complementary hole centers ($V_{n}$), which have an absorption band maximum in the vacuum UV region [17]. During annealing, some defects recombine while others participate in aggregation processes, as indicated by the increased absorption in the spectral region associated with $F_{n}$ centers. Similar defects are observed under irradiation with pulsed electron beams, although the efficiency of defect creation and the aggregation processes are less pronounced.

In our case, ion tracks are formed under irradiation with 150 MeV ^84^Kr ions, and hillocks also appear on the surface [58]. Track formation occurs under irradiation with both 150 MeV ^84^Kr and 2640 MeV ^238^U ions, with hillock structures also forming on the surface [17,57,58].

### 3.3. The F-Type Center Annealing Analysis

In this study, the theoretical model of stepwise annealing in *MgF*_2_ crystals [59] is employed, which is based on the phenomenological theory of diffusion-controlled recombination kinetics of single-electron centers in irradiated ionic crystals [60,61]. This phenomenological theory has previously been applied to the annealing of *F* centers under gamma [62] and neutron [63] irradiation of *MgF*_2_ crystals, as well as to the thermal annealing and transformation of $F_{2}$ centers.

In reference [59], the phenomenological theory was further developed, demonstrating how it is fitting to the experimental un annealed curves allows one to extract two control parameters: the migration energy of interstitial ions $E_{a}$ and the pre-exponential factor $X=\frac{N_{0}a_{0}D_{o}}{\beta }$, where $N_{0}$ is the initial defect concentration, $a_{0}$ is the recombination radius, $D_{o}$ is the diffusion pre-exponential factor, and β is the heating rate.

**Figure 6:**
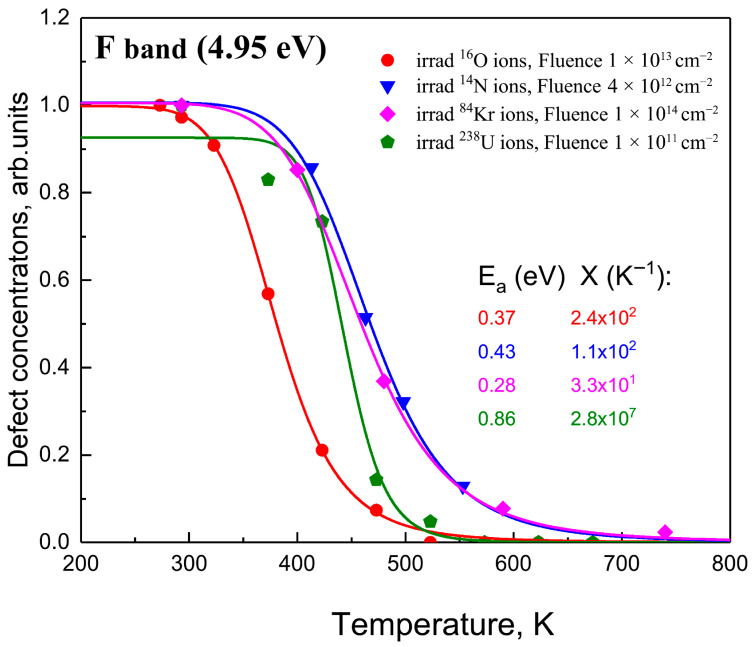
Dependence of F-center concentration on the temperature of intermediate annealing steps for LiF crystals irradiated with oxygen, nitrogen, krypton, and uranium ions at various fluences.

**Figure 7:**
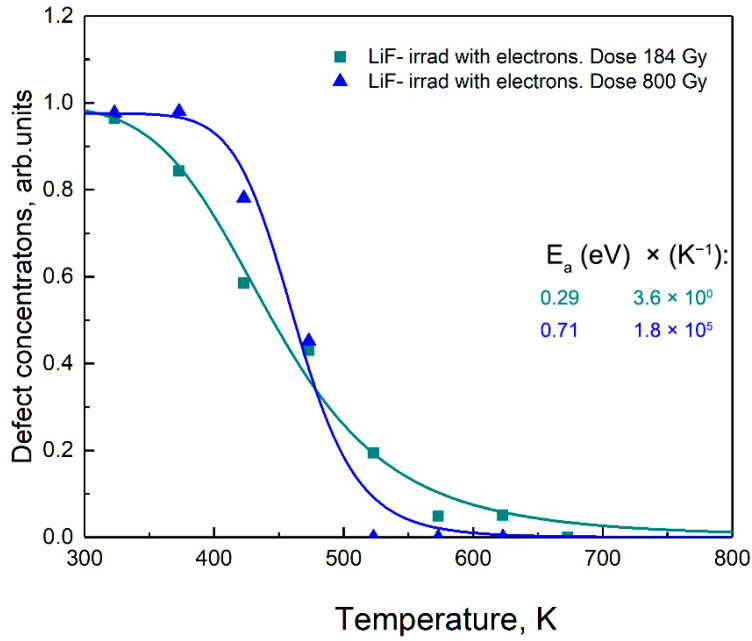
Dependence of F-center concentration on the temperature of intermediate annealing steps for LiF crystals irradiated with electron beams.

The results of the theoretical analysis of F-center annealing in LiF are presented in Figure 3 and Figure 4 and Table 3 is shown in Figure 6 and Figure 7.

**Figure 8:**
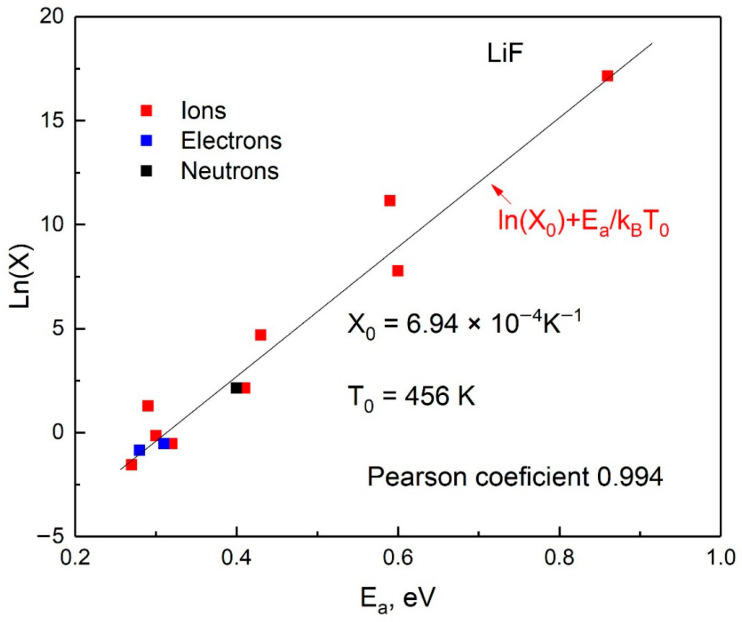
Correlations for LiF.

From Table 3, a correlation was observed between the activation energy $E_{a}$ and the pre-exponential factor $X\left(E_{a}\right)$, where $X\left(E_{a}\right)$ is no longer constant but shows very good agreement with an exponential function of $E_{a}$, as illustrated in Figure 8.

The results obtained in this study demonstrate a wide variation in the parameters $E_{a}$ and X depending on the irradiation dose. An increase in the electron irradiation dose from $1.84\times 10^{2}$ Gy → $8\times 10^{2}$ Gy → $0.5\times 10^{6}$ Gy leads to a sharp increase in $E_{a}$ from 0.29 eV ($X=3.6\times 10^{0}\;K^{-1}$) to 1.07 eV ($X=4.5\times 10^{6}\;K^{-1}$). Similar effects are observed under irradiation with light ions, such as 28 MeV ^16^O and 130 MeV ^12^C. The increase in diffusion energy is accompanied by an orders-of-magnitude increase in the pre-exponential factors. These results confirm the conclusion drawn in [59] that the observed dependence of the diffusion parameter on irradiation dose, as well as the correlation between $E_{a}$ and X, is associated with the increasing disorder of the material at high irradiation doses.

A strong correlation is observed between the activation energy $E_{a}\;$ and the pre-exponential factor X, where X is no longer constant but agrees very well with a potential (power-law) function of $E_{a}$. Indeed, with increasing irradiation dose, both the migration energy $E_{a}\;$ and the pre-exponential factor X decrease simultaneously, resulting in an effective increase in the defect diffusion rate.

A similar correlation has been reported for several irradiated ionic materials—Al_2_O_3_ [64,65,66,67], MgO [68], and MgF_2_—which suggests that this phenomenon may be rather widespread [59]. Indeed, such a regularity has been experimentally observed even in materials such as MgAl_2_O_4_ [69], diamond [70] and theoretically predicted in SiO_2_ [71].

## 4. Conclusions

Irradiation of LiF with swift heavy ions (N, O, Kr, U) predominantly produces F centers together with aggregate electron centers (F_n_) and their complementary hole defects, whereas under the electron-irradiation conditions explored, the defect spectrum is dominated by F centers (no F_n_ bands resolved at 184 Gy). Thermal analysis shows that the principal relaxation of radiation damage proceeds above~470 K, with a sharp decrease in the F-band amplitude and integral intensity between ~473–523 K, followed by near-complete recovery at higher temperatures depending on the projectile: ~590–623 K for O and N, ~723 K for U, and up to ~850 K for Kr. In the mid-temperature regime (~600 K), defect migration fosters aggregation reactions and the emergence of lithium/impurity colloids; for high-electronic-stopping projectiles (Kr, U), this accompanies ion-track formation and surface hillocks. 

Kinetic analysis of stepwise annealing yielded the activation energy E_a_ and the corresponding pre-exponential factor for the dominant recovery process. Across irradiation types, these parameters span a broad range, e.g., E_a_ ≈ 0.28–0.43 eV for 150 MeV Kr, 28 MeV O, and 23 MeV N; ~0.86 eV for 2640 MeV U; and ~0.29–1.07 eV for electron-irradiated samples depending on energy/dose. A robust correlation between E_a_ and the pre-exponential factor is observed, evidencing dose-dependent changes in defect transport consistent with disorder-controlled diffusion; this trend holds across LiF datasets and aligns with behavior reported for other ionic crystals.

Overall, the results define practical temperature windows for recovery of LiF after irradiation, clarify how electronic stopping governs defect aggregation versus recombination, and establish quantitative kinetic parameters (E_a_ and pre-exponential factor) for defect annealing that can guide post-irradiation thermal treatments and the controlled tuning of color-center concentration in LiF-based optical and dosimetric applications.

## Figures and Tables

**Table 1:** Absorption band maxima of F-type centers in LiF crystals. The absorption maximum $\lambda_{max}$ at a certain temperature and the annealing temperature $T_{a}$ and models are indicated [17,28,30,31,33,40,41,42].

Center	Model	Absorption Maximum	
		$\lambda_{max}$ (nm)	$\lambda_{max}$ (eV)
F	$V_{a}^{+}+e^{-}$	250 (300 K)	4.96
$F_{2}$	${2V}_{a}^{+}+2e^{-}$	445 (300 K)	2.79
$F_{2}^{+}$	$2V_{a}^{+}+e^{-}$	625	1.98
$F_{2}^{-}$	${2V}_{a}^{+}+{3e}^{-}$	950	1.31
$F_{3}\left(R_{1}\right)$	${3V}_{a}^{+}+{3e}^{-}$	317 (300 K)	3.91
$F_{3}\left(R_{2}\right)$		377 (300 K)	3.29
$F_{3}^{+}$	${3V}_{a}^{+}+{2e}^{-}$	442	2.81
$F_{3}^{-}$	${2V}_{a}^{+}+{4e}^{-}$	820	1.51
$F_{4}$ ($N_{1}$)	$4V_{a}^{+}+{4e}^{-}$	518 (300 K)	2.39
$F_{4}$ ($N_{2}$)		540 (300 K)	2.3

**Table 2:** Radiation parameters of ^16^O, ^14^N, ^84^Kr, and ^238^U ions in LiF crystals.

Ion	Energy (MeV)	Range (µm)	$\frac{E}{R}$, KeV/nm	Electronic Stopping Power, $S_{e}$ KeV/nm	Nuclear Stopping Power, $S_{n}$ KeV/nm
^16^O	28	14.91	1.88	1.76	0.0013
^14^N	23	14.13	1.63	1.52	0.0011
^84^Kr	150	17.76	8.45	12.12	0.022
^238^U	2640	94.02	28.08	0.03	0.0026
^16^O	28	14.91	1.88	1.76	0.0013

**Table 3:** Obtained values of the migration energy $E_{a}\;$ of interstitial ions and the pre-exponential factor X for different types of irradiation and various fluences.

**№**		Experiment			Modeling	
	**Methods**	Source	Dose (Gy)	Ref.	$E_{a}$ (eV)	$X\left(K^{-}\right)$
1	Optical absorption	O ions 28 MeV Fluence $1\times 10^{13}$ cm^−2^	$1.13\times 10^{7}$	This work	0.37	$2.4\times 10^{2}$
2	Optical absorption	N ions 23 MeV Fluence $4\times 10^{12}$ cm^−2^	$3.9\times 10^{7}$	This work	0.43	$1.1\times 10^{2}$
3	Optical absorption	Kr ions 150 MeV Fluence $1\times 10^{14}$ cm^−2^	$5.13\times 10^{5}$	This work	0.28	$3.3\times 10^{1}$
4	Optical absorption	U ions 2640 MeV Fluence $1\times 10^{11}$ cm^−2^	$1.7\times 10^{7}$	This work	0.86	$2.8\times 10^{7}$
5	Optical absorption	Electrons 250 KeV	$1.84\times 10^{2}$	This work	0.29	3.6
6	Optical absorption	Electrons 250 KeV	$8\times 10^{2}$	This work	0.71	$1.8\times 10^{5}$
7	Optical absorption	C ions 130 MeV Fluence $1\times 10^{12}$ cm^−2^	$3.34\times 10^{8}$	[54]	0.32	$5.8\times 10^{-1}$
8	Optical absorption	C ions 130 MeV Fluence $1\times 10^{11}$ cm^−2^	$3.34\times 10^{9}$	[54]	0.60	$2.4\times 10^{3}$
9	Optical absorption	Electrons 10 MeV	$0.5\times 10^{6}$	[54]	1.07	$4.5\times 10^{6}$
10	Optical absorption	Au ions 709 MeV Fluence $1\times 10^{12}$ cm^−2^	$1.16\times 10^{6}$	[54]	0.31	$5.8\times 10^{-1}$
11	Optical absorption	Au ions 709 MeV Fluence $1\times 10^{11}$ cm^−2^	$1.16\times 10^{7}$	[54]	0.28	$4.3\times 10^{-1}$
12	Optical absorption	Xe ions 800 MeV Fluence $3\times 10^{11}$ cm^−2^	$2.97\times 10^{7}$	[54]	0.30	$8.5\times 10^{-1}$
13	Optical absorption	Pb ions 2300 MeV Fluence $5\times 10^{10}$ cm^−2^	$7.19\times 10^{8}$	[54]	0.27	$2.1\times 10^{-1}$
14	Optical absorption	U ions 2640 MeV Fluence $5\times 10^{10}$ cm^−2^	$8.53\times 10^{8}$	[54]	0.41	8.6
15	EPR	Neutron irradiation $1\times 10^{16}$ n/cm^−2^	$12.6\times 10^{6}$	[55]	0.40	8.5

## Data Availability

The original contributions presented in this study are included in the article. Further inquiries can be directed to the corresponding authors.
